# Differentiation of Autoimmune Pancreatitis From Pancreatic Ductal Adenocarcinoma by Diffusion-Weighted Magnetic Resonance Imaging With Weighted Diffusion Subtraction

**DOI:** 10.7759/cureus.81438

**Published:** 2025-03-29

**Authors:** Jun Woo, Katsuhiro Sano, Koichi Oshio, Akiyoshi Suzuki, Hiroyuki Isayama, Akio Saiura, Yuki Fukumura, Koji Kamagata, Ryohei Kuwatsuru, Shigeki Aoki

**Affiliations:** 1 Department of Radiology, Jikei University School of Medicine, Tokyo, JPN; 2 Department of Radiology, Juntendo University Graduate School of Medicine, Tokyo, JPN; 3 Department of Gastroenterology, Juntendo University Graduate School of Medicine, Tokyo, JPN; 4 Department of Hepatobiliary‐Pancreatic Surgery, Juntendo University Graduate School of Medicine, Tokyo, JPN; 5 Department of Human Pathology, Juntendo University Graduate School of Medicine, Tokyo, JPN

**Keywords:** apparent diffusion coefficient (adc), autoimmune pancreatitis (aip), diffusion-weighted image, pancreatic ductal adenocarcinoma, weighted diffusion subtraction

## Abstract

Purpose

Weighted diffusion subtraction (WDS) is a new imaging technique derived from a pair of diffusion-weighted images (DWIs) using low and high b values. This study assesses the diagnostic performance of WDS in distinguishing pancreatic ductal adenocarcinoma (PDAC) from autoimmune pancreatitis (AIP) by comparing the efficacy of DWI alone compared to DWI with WDS.

Methods

The patient cohort consisted of 10 patients diagnosed with AIP and 28 patients diagnosed with PDAC. Two blinded radiologists reviewed the MRI images and assessed their diagnostic accuracy and confidence levels. Differences between groups A (DWI alone) and B (DWI added WDS) were assessed, and a receiver operating characteristic (ROC) curve analysis was performed. Inter-reader agreement was analyzed using weighted κ statistics, and apparent diffusion coefficient (ADC) values were calculated for both AIP and PDAC.

Results

In diagnostic accuracy, Group B demonstrated significantly higher area under the curve (AUC) values than Group A for both readers. The inter-reader agreement was evaluated as “substantial” in both groups. The mean confidence scores for Group B were significantly higher than those for Group A for both readers.

Conclusion

The addition of WDS to DWI may enhance the visual differentiation between AIP and PDAC, potentially improving diagnostic accuracy and confidence.

## Introduction

Autoimmune pancreatitis (AIP) is a distinct form of pancreatitis characterized by inflammation due to an autoimmune response [1]. Yoshida et al introduced the term AIP in 1995 to describe steroid-responsive mass-forming pancreatitis with elevated autoantibodies [2, 3]. The exact incidence and prevalence of AIP remain unknown; however, it accounts for 5-6% of all patients with chronic pancreatitis [3, 4]. In Japan, where AIP is more prevalent than in Western countries, the overall prevalence is 4.6 per 100,000 people, with an annual incidence of 1.4 per 100,000 people [5].

The focal form of AIP is typically in the pancreatic head, appearing as an enlargement or mass-like structure with delayed enhancement after the administration of a contrast agent. Occasionally, AIP may present as a multifocal mass and generally the apparent diffusion coefficient (ADC) value of AIP was lower than that of normal pancreatic tissue and pancreatic cancer, which causes restricted diffusion [6]. Both type 1 and type 2 AIP can appear similar on imaging. Recent studies indicate that type 2 AIP are more likely to be focal (up to 85%) [7]. Patients with type 1 AIP may exhibit a higher degree of main pancreatic duct dilation, possibly due to inflammatory infiltrates and fibrosis compressing the duct [8]. Differentiating AIP from pancreatic ductal adenocarcinoma (PDAC) is critical, as both can present as pancreatic masses.

Pancreatic cancer is one of the most intractable cancers owing to its challenging early detection. According to the Global Cancer Observatory (GLOBOCAN) 2020, an estimated 495,773 patients were newly diagnosed with pancreatic cancer in 2020 worldwide, ranking pancreatic cancer 12th among all malignant tumors [9]. An estimated 466,003 deaths were attributed to pancreatic cancer in 2020, resulting in pancreatic cancer ranking 7th among all malignant tumors [9]. Both environmental and genetic factors likely contribute to pancreatic cancer development. Currently, early-stage surgical resection is the only effective treatment option. Therefore, early diagnosis and timely surgical intervention are critical for improving the outcomes of patients with pancreatic cancer [9].

Patients with AIP often respond well to steroid therapy, while those with PDAC require surgery and/or chemotherapy [6,10,11]. Differentiating AIP from PDAC is crucial for providing appropriate treatment and preventing unnecessary surgery [12]. Mass-forming AIP may exhibit imaging features similar to PDAC [13]. Some studies have reported significantly lower ADC values in patients with AIP compared to those with PDAC [6,12]. However, visually distinguishing PDAC from mass-forming AIP on ADC maps remains challenging.

Weighted diffusion subtraction (WDS) is a new image from a pair of DWI obtained with low and high b values, using a pixel-by-pixel calculation:

S(x, y)＝S0(x, y)exp (－ΔbD_thres_)－S1(x, y) ＝S0(x, y)exp (－ΔbD_thres_)－S0(x, y) exp (－ΔbADC(x, y)) [14]

where *S* is the signal intensity of the new image, *S0* is that of the image obtained with the low b value, and *S1* is that of the image acquired with the high b value. *Dthres* is the threshold value of the diffusion coefficient at which the sign of the signal of the new image is inverted, and *Δb* is the difference between the b values of the two diffusion-weighted images (DWIs). Because the exponential term with *Dthres* is just a constant, this calculation is a simple linear combination of the two DWIs, and the method is tentatively called WDS. The contrast of the new image is similar to that of the T2-weighted image (S0) but with the signs of the pixels with small ADC values inverted [15]. WDS is an image processing technique that uses a pair of low- and high-b-value DWI to create a single image containing clinically relevant information without the ambiguity of the T2 shine-through [15]. Each pixel in the WDS image takes a positive or negative value depending on whether its ADC value is below the threshold ADC value. This simplifies image evaluation, allowing for clear visual evaluation of areas with ADC values below the threshold. This study evaluates the improvement in the diagnostic ability of WDS in differentiating PDAC from mass-forming AIP by comparing DWI and ADC map to DWI and ADC map with WDS.

## Materials and methods

Patients

**Figure 1:**
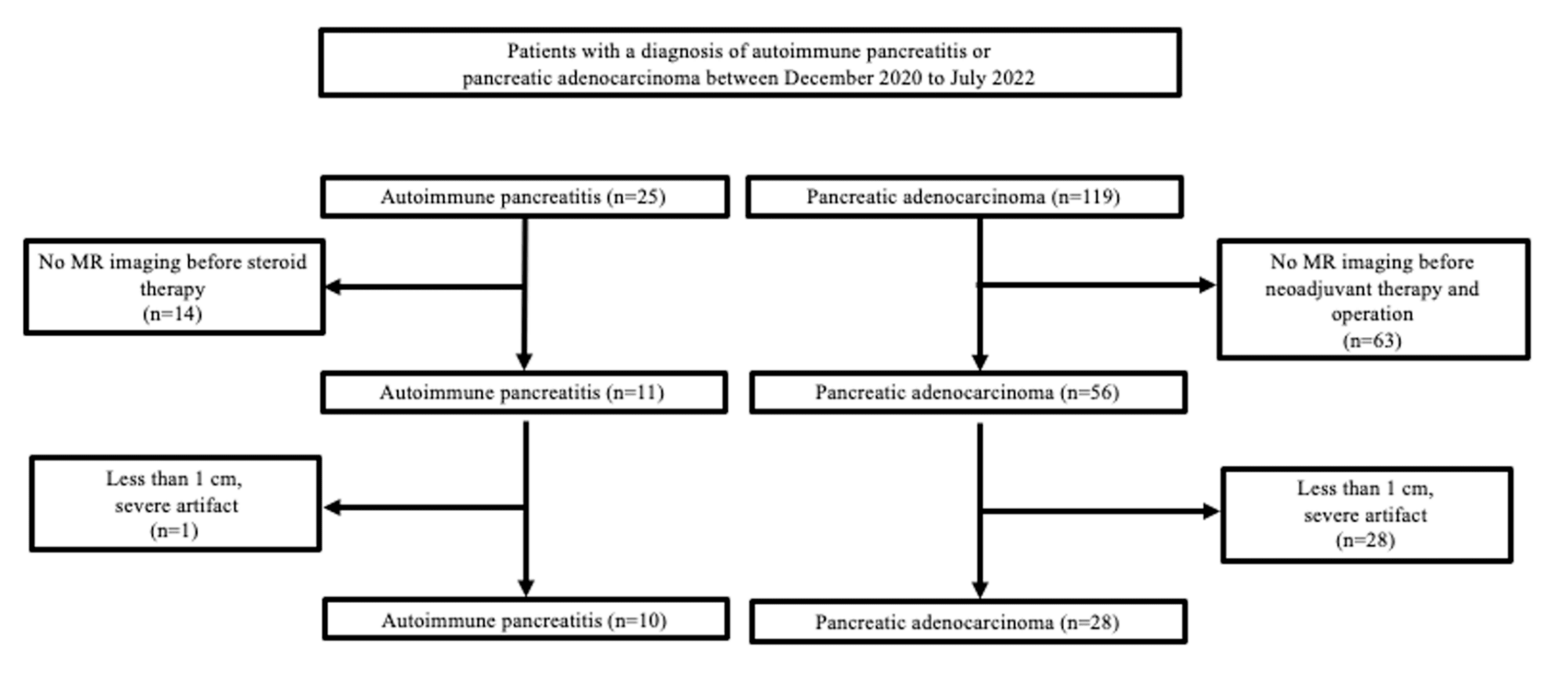
Flow diagram showing the selection of the study population

This study was approved by the institutional review board of Juntendo University School of Medicine for Biomedical Research (E23-0166), and the requirement for informed consent was waived due to its retrospective nature. From December 2020 to July 2022, we enrolled 25 patients with AIP diagnosed according to the 2018 Japanese Clinical Diagnostic Criteria for Autoimmune Pancreatitis and 119 patients with PDAC, confirmed by pathology. Fourteen patients with AIP and 63 patients with PDAC who had not undergone MRI before therapy were excluded, along with one patient with AIP and 28 patients with PDAC whose lesion size was less than 1 cm or who had severe motion artifacts on MR images. Therefore, the final number of patients included in this study was 10 patients with AIP and 28 patients with PDAC (Figure 1).

Imaging technique

MRI scans were performed using the Intera Achieva 3.0T and Ingenia 1.5T (Philips Healthcare, Best, The Netherlands), MAGNETOM Prisma 3.0T, MAGNETOM Skyra 3.0T, MAGNETOM Avanto (Siemens, Erlangen, Germany), and Vantage Centurian 3.0T (Canon Medical Systems, Tochigi, Japan). All transverse MR images were obtained with a section thickness of 6 mm and a 0.6 mm intersection gap. Respiratory-triggered DWIs (TR/TE, 1000-7000/ 46-81 ms: field of view, 36 × 36 cm; b-value, 0, 800 (1.5T), and 1000 (3.0T) s/mm^2^) were obtained from all patients. Additional imaging parameters are listed in Table 1.

**Table 1:** Parameters of diffusion-weighted imaging (DWI) FB: free breath, RT: respiratory triggered, T: telsa, NEX: number of excitations, FOV: field of view

Parameters of DWI	
TR (ms)	(FB) 7000, (RT) 970-1340
TE (ms)	(FB) 81.65, (RT) 46-68
Matrix	128
NEX	2
Thickness/spacing	6/0
FOV (cm×cm)	36
b value (s/mm^2^)	(3T) 1000, (1.5T) 800

Imaging assessment

The case sets were prepared by the author. Two radiologists with six and 19 years of post-training experience in abdominal imaging independently reviewed images from DWI and ADC maps (Group A) and DWI and ADC maps with WDS (Group B) while blinded to clinical information and pathological characteristics. Each radiologist reviewed both groups independently for all patients. For Group A, the radiologists examined the anatomical locations, imaging findings, and strength of diffusion restriction of the lesion on the axial DWI (low b and high b) and the ADC map. In Group B, in addition to those in Group A, WDS images were examined and evaluated simultaneously.

For qualitative assessment, the reviewer evaluated the imaging criteria used to distinguishing between AIP and PDAC as follows: findings suggestive of AIP included marked diffusion restriction, pancreatic enlargement, absence of main pancreatic duct dilation, and a homogeneous mass. By contrast, findings suggestive of PDAC included mild diffusion restriction, caudal pancreatic atrophy, dilation of the distal main pancreatic duct, and a heterogeneous mass.

Regarding diagnostic accuracy, diffusion restriction was evaluated using DWI and ADC maps in Group A, and WDS was also evaluated in Group B. Furthermore, cases meeting two or more of the imaging diagnostic criteria for each disease were classified on a three-point scale as "suspected AIP" or "suspected PDAC," respectively. Cases that did not meet these criteria were classified as "undetermined." In cases that met the imaging criteria for both diseases and in which it was difficult to determine the diagnosis as either AIP or PDAC, we also classified them as "undetermined."

Regarding the degree of confidence, the reader subjectively assessed 'Indistinguishable,' 'Slightly distinguishable,' or 'Distinguishable' based on how confidently they could differentiate between the two using the image findings. The ADC cut-off value (×10^-3^ mm^2^/s) was determined to be 1.274 (95% CI: 0.732-1.816) using receiver operating characteristic (ROC) analysis. The cut-off value was subsequently applied to generate WDS images. ADC values (×10^-3^ mm^2^/s) were measured on ADC maps by placing regions of interest (ROIs) of approximately 25-100 mm^2^ within the lesions and the surrounding normal pancreatic parenchyma. The WDS tumor-to-pancreatic parenchyma signal intensity ratio was calculated by dividing the signal intensity of the lesion by that of the adjacent normal pancreatic tissue on the WDS images, using the same ROIs as for the ADC measurements. The WDS application used in this study allows the use of black-and-white inverted images of the WDS, and the image evaluator also assesses the black-and-white inverted images as appropriate.

Statistical analysis

All statistical analyses were performed using IBM SPSS Statistics for Windows, Version 29.0 (released 2013, IBM Corp., Armonk, NY). Chi-square test was used for nominal and ordinal variables. Data for continuous variables were expressed as mean ± standard deviation and assessed using *t-test*. Diagnostic accuracy and the reader confidence score of both groups were assessed using the Mann-Whitney U test. The area under the curve (AUC) for diagnostic accuracy was calculated using ROC curve analysis. The inter-reader agreement was analyzed using *weighted κ statistics*. The weighted kappa value was interpreted as follows: <0, No agreement; 0.00-0.20, slight agreement; 0.21-0.40, fair agreement; 0.41-0.60, moderate agreement; 0.61-0.80, substantial agreement; and 0.81-1.00, almost perfect.

The ROI in the WDS images was placed within the lesions and the normal pancreatic parenchyma surrounding them as extensively as possible. The ratio of the signal intensity of the lesion to that of the pancreatic parenchyma on WDS images was calculated and recorded. The ratio of signal intensities in the lesion to that in the pancreatic parenchyma on WDS images was calculated using the t-test. Statistical significance was set at *p *< 0.05.

## Results

The study analyzed DWI and ADC map with and without the inclusion of WDS for differentiating AIP from PDAC. The patient cohort included 10 cases of AIP and 28 cases of PDAC. Demographic and clinical characteristics, including age, sex, lesion size, or pancreatic location, showed no significant differences between the two groups (Table 2).

**Table 2:** Demographic and clinical characteristics of the study patients. Chi-square test was used for nominal and ordinal variables. Data for continuous variables were expressed as mean ± standard deviation and assessed using *t-test*. Statistical significance was set at p < 0.05. IgG4: immunoglobulin G4, CA19-9: carbohydrate antigen 19-9

Parameters	AIP (n = 10)	PDAC (n = 28)	Statistical Analyses / *t/chi-square value*	p-value
Age (years)	69.9 ± 9.8	65.6 ± 9.7	t-test / 0.889	0.591
Sex (male/female)	7/3	15/13	chi-square test / 0.816	0.336
Maximum lesion size (mm)	34.2 ± 18.2	25.2 ± 9.5	t-test / -1.978	0.056
Pancreatic location lesions			chi-square test / 0.368	0.832
Head	50% (5/10)	61% (17/28)		
Body	30% (3/10)	25% (7/28)		
Tail	20% (2/10)	14% (4/28)		
Laboratory data				
IgG4 (> 135mg/dl)	937.3 ± 1335.3	NA		
CA19-9 (> 37U/ml)	NA	224.5 ± 424.7		

**Figure 2:**
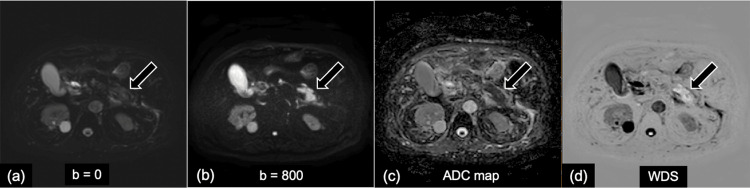
Representative case of a man in his 70s with focal-type autoimmune pancreatitis (AIP) in pancreatic tail. (a) diffusion-weighted image (DWI) b value = 0 s/mm^2^, (b) DWI b value = 800 s/mm^2^, (c) apparent diffusion coefficient (ADC) map, (d) weighted diffusion subtraction (WDS) image. (a, b) On the DWI (b = 0, 800 s/mm^2^), an irregularly shaped mass located in the tail of the pancreas shows high signal intensity compared with the normal pancreas parenchyma. (d) The WDS image shows the mass with high-signal-intensity "white-out."

**Figure 3:**
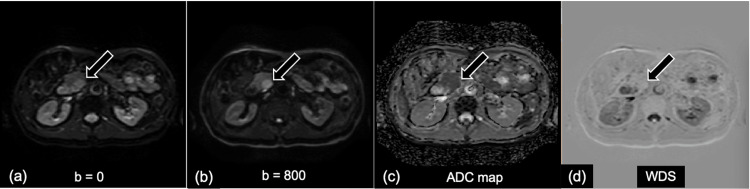
A representative case of a man in his 60s with pancreatic ductal adenocarcinoma (PDAC) in the pancreatic head. (a) diffusion-weighted image (DWI) b value = 0 s/mm^2^, (b) DWI b values = 800 s/mm^2^, (c) apparent diffusion coefficient (ADC) map, (d) weighted diffusion subtraction (WDS) image. (a, b) On the DWI (b = 0, 800 s/mm^2^), an irregularly shaped mass located in the head of the pancreas shows high signal intensity compared with the normal pancreas parenchyma. (d) The WDS image shows the mass with peripheral slightly high signal intensity and central low intensity.

Representative DWI, ADC map, and WDS images used for visual and quantitative assessment in AIP and PDAC are presented in Figures 2-3.

**Figure 4:**
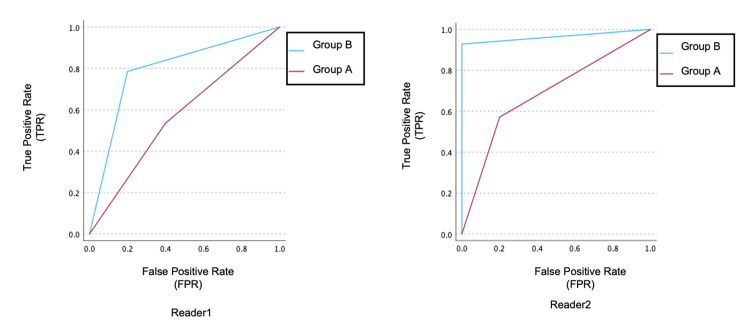
Area under the curve (AUC) and inter-reader agreement of diagnostic accuracy Readers 1 and 2 with six and 19 years of post-training experience in abdominal imaging independently reviewed images from the diffusion-weighted image (DWI) and apparent diffusion coefficient (ADC) map (group A) and DWI and ADC map with weighted diffusion subtraction (WDS) (group B).

The area under the curve (AUC) for diagnostic accuracy in Group A (DWI and ADC map) was 0.814 for Reader 1 and 0.886 for Reader 2, whereas in Group B (DWI and ADC map with WDS), the AUC increased to 0.904 for Reader 1 and 0.979 for Reader 2, indicating a statistically significant improvement (p < 0.001) (Figure.4).

**Figure 5:**
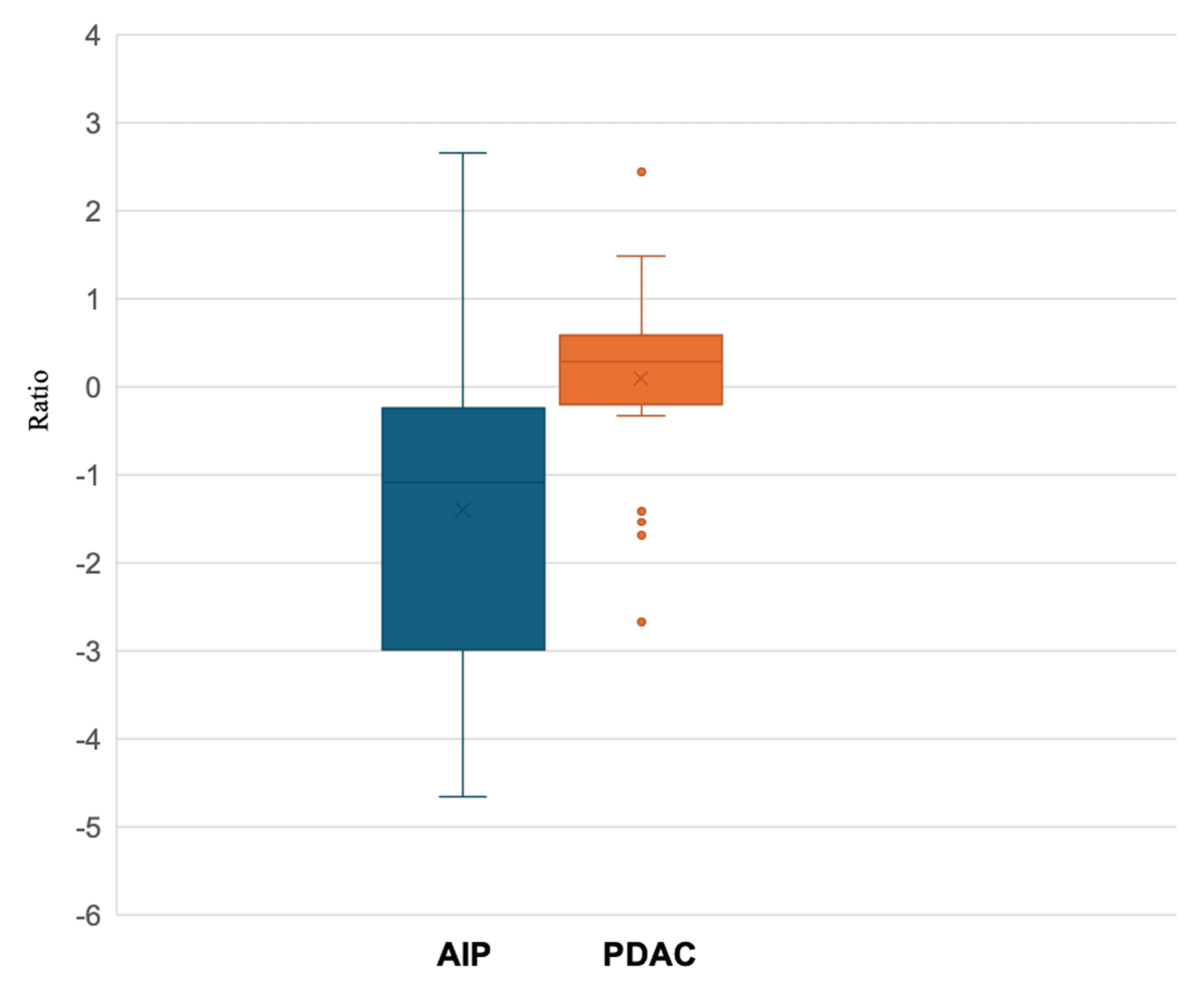
Weighted diffusion subtraction (WDS) tumor/pancreatic cancer parenchyma ratio for autoimmune pancreatitis (AIP) and pancreatic ductal adenocarcinoma (PDAC) lesions Center lines show medians, box limits indicate the 25th and 75th percentiles, and whiskers extend 1.5-times interquartile range from the 25th to 75th percentiles.

Based on the three-point diagnostic confidence scale, 16 and 15 cases were classified as "undetermined" in Group A by Reader 1 and Reader 2, respectively. Among these, 14 cases for Reader 1 and nine cases for Reader 2 were correctly diagnosed in Group B. Inter-reader agreement, assessed using weighted kappa (κ) statistics, was higher in Group B (κ = 0.743) than in Group A (κ = 0.676), indicating improved consistency between radiologists. In addition, confidence scores significantly increased in Group B compared to Group A (Reader 1: 1.87 vs. 2.32; Reader 2: 1.82 vs. 2.71; p < 0.001), suggesting that WDS contributed to enhanced diagnostic confidence. Moreover, the lesion-to-pancreatic parenchyma signal intensity ratio on WDS images was significantly lower in AIP than in PDAC (p = 0.009), further supporting the utility of WDS in disease differentiation (Figure 5).

## Discussion

This study demonstrated that adding WDS to DWI improves the visual differentiation of AIP from PDAC. Compared to Group A using DWI and ADC map, Group B with added WDS showed a significant improvement in diagnostic accuracy, as measured by the AUC. The AUCs for differentiating AIP from PDAC using ADC maps were equivalent to those reported in previous studies [6,12].

Clinically, AIP and PDAC share symptoms such as painless jaundice, new-onset diabetes mellitus, and weight loss, often with a concurrent pancreatic mass. Consequently, distinguishing AIP from PDAC remains challenging [16,17]. Accurate differentiation is crucial, as misdiagnosis may lead to unnecessary surgery [16-18]. While typical AIP cases characterized by diffuse pancreatic enlargement and a capsule-like rim on computed tomography (CT) or MRI are relatively easy diagnose, focal or segmental AIP cases can be difficult to distinguish from pancreatic cancer cases, even with CT, MRI, and 18F-fluorodeoxyglucose positron emission tomograph (FDG-PET) [6,17,19-24].

DWI is a functional diagnostic imaging method that allows the visualization of the movement of water molecules in tissues. Generally, malignant tumors have a lower ADC than normal tissues and show clear and high signals on DWI. Both PDAC and AIP appeared as high-signal-intensity lesions on DWI. Several previous studies have evaluated the role of the DWI and ADC map in obtaining a differential diagnosis of AIP and pancreatic cancer [1,6,12,25]. In these studies, the reported ADC values for AIP were significantly lower than those for pancreatic cancer, chronic alcoholic pancreatitis, and the normal pancreas [1,6,12,25]. However, visually distinguishing AIP from pancreatic ductal adenocarcinoma is challenging without precise ADC measurements, raising concerns for clinical applications. Qualitative assessment is particularly difficult for ADC maps due to low tissue contrast and poor depiction of anatomical structures [26,27]. Sato et al. reported that the contrast between prostate cancer and normal prostate tissue was significantly higher in WDS images than in ADC maps, and a PI-RADS DWI score based on WDS images achieved better diagnostic accuracy and higher inter-reader agreement for assessing prostate cancer than the traditional PI-RADS DWI score based on ADC [26,27].

Therefore, this study evaluated whether the addition of WDS to DWI could improve the differential diagnostic ability for AIP and PDAC. Consequently, adding the WDS improved diagnostic accuracy and confidence and increased inter-reader agreement. These findings suggest that WDS overcomes the limitations of DWI and provides clearer images, facilitating the differentiation of AIP from PDAC.

With WDS, the signal intensity of AIP can be displayed as a negative value based on a threshold, visually "whiting out" the image and improving visibility. The use of WDS has been suggested to enhance diagnostic confidence. With clearer images, readers may gain more confidence in their diagnoses, potentially reducing the risk of misdiagnosis.

The inter-reader agreement in diagnostic scoring was “substantial,” indicating consistent evaluations without significant variability between readers. Adding WDS reduces subjective interpretation variability, allowing for more objective evaluations.

This study has several limitations. The first limitation is that this was a retrospective study conducted at a single center with a multi-unit MR design. Since appropriate determination of the ADC threshold is crucial for enhancing the diagnostic performance of WDS, future studies should aim to establish this threshold based on a larger cohort and validate its effectiveness through prospective investigations.

The second limitation is that the number of AIP patients examined for mass-forming AIP was small. In addition, the degree of lymphoplasmacytic infiltration could not be evaluated because some AIP cases lacked pathological evidence. However, according to the "2018 Japanese Clinical Diagnostic Criteria for Autoimmune Pancreatitis," which we used in our study, a definitive diagnosis of focal AIP can be made even without histopathological confirmation, provided that the following are present: localized pancreatic enlargement on imaging, narrowing of the main pancreatic duct on ERCP, hyper-IgG4emia (>135 mg/dL) based on serological findings, IgG4-related extrapancreatic lesions (such as sclerosing cholangitis, sclerosing dacryoadenitis/sialadenitis, retroperitoneal fibrosis, or interstitial nephritis) based on clinical and imaging findings, and a positive therapeutic response to steroid treatment. All cases without histopathological confirmation in this study were diagnosed based on these criteria. We also included lesions slightly larger than 3 cm, as defined by the Hagga criteria in our sample cases. Therefore, further prospective studies are needed, involving more patients with mass-forming AIP. However, this is the first report on the usefulness of WDS to distinguish AIP from pancreatic ductal adenocarcinoma. 

The third limitation is that some cases in Group A may have been partially diagnosable. However, slightly less than half of the cases could not be diagnosed in Group A but were correctly diagnosed in Group B, suggesting that the addition of WDS may be beneficial.

The fourth limitation is that this study did not employ noise reduction techniques, such as deep learning-based reconstruction, which might have improved image quality and may also be important for accurate ADC threshold determination. This warrants further investigation. It should be noted that the ADC value was used solely to set the threshold for WDS, and no direct quantitative evaluation was performed.

## Conclusions

This study found that the addition of WDS to DWI may enhance the visual differentiation between AIP and PDAC, potentially improving diagnostic accuracy and confidence. To achieve this, creating ADC maps with minimal artifacts and setting accurate thresholds are essential steps for maximizing the diagnostic utility of WDS. This combination may support more accurate diagnoses and better treatment decisions for mass-forming AIP, thereby reducing unnecessary surgeries.
